# In-clinic vitreous biopsy peel pack technique

**DOI:** 10.1186/s40942-025-00639-8

**Published:** 2025-02-10

**Authors:** Charles DeBoer, Cindy Zhao, Prithvi Mruthyunjaya, Vinit B. Mahajan, Karen M. Wai, Steven R. Sanislo

**Affiliations:** 1 Molecular Surgery Laboratory, Stanford University, 2452 Watson Court, Palo Alto, CA 94304 USA; 2 Department of Ophthalmology, Byers Eye Institute, Stanford University, 2452 Watson Court, Palo Alto, CA 94304 USA; 3 Veterans Affairs Palo Alto Health Care System, 3801 Miranda Ave, Palo Alto, CA 94304 USA

**Keywords:** Vitreous biopsy, Endophthalmitis, Vitrectomy, Office-based vitrectomy, Uveitis, Masquerade syndrome

## Abstract

**Background:**

Vitreous biopsy is a common technique used to guide management of acute endophthalmitis and help differentiate between infectious and inflammatory conditions. Currently, in-clinic vitreous biopsy is performed with a 25-gauge needle, without the ability to cut vitreous, potentially leading to reduced diagnostic yield. Recent work demonstrated the ability to perform vitreous biopsy with an off-the-shelf vitreous cutter. However, this was limited by complexity of assembly. Here, a technique using a single peel pack vitrectomy cutter is demonstrated for in-clinic vitreous biopsy.

**Methods:**

A 25-gauge vitreous cutter is opened from a peel pack. The drive line is identified, cut to length, and attached to a 10 mL syringe. A 1 mL syringe is attached to the aspiration line. After a trocar is used to place a cannula in the pars plana, the vitreous cutter is introduced into the eye. Cutting is performed by an assistant actuating the 10 mL syringe while the surgeon aspirates from the 1 mL syringe. After sample is collected, antimicrobials are injected if required and the cannula is removed.

**Results:**

A peel pack technique simplifies assembly for an in-clinic vitreous biopsy using a manually actuated cutter.

**Conclusion:**

We present a novel, improved, and simplified technique for vitreous tap using a vitreous cutter provided in a single peel pack, actuated by a single syringe with minimal assembly prior to use. This technique may be more accessible for clinicians than prior techniques and does not require a surgical console.

**Supplementary Information:**

The online version contains supplementary material available at 10.1186/s40942-025-00639-8.

## Background

Vitreous biopsy is a commonly performed procedure used to guide management of acute endophthalmitis to differentiate inflammatory and infectious processes and to evaluate for malignancy [1, 2]. Vitreous biopsy can be performed in the clinic using a vitreous tap with a standard needle, or with pars plana vitrectomy, either in an office setting or in the operating theater. In-clinic vitreous biopsy offers the advantage of speed such as in the case of acute endophthalmitis and does not require complex anesthesia. However, needle biopsy can have a high range of dry taps, with studies reporting up to 35% [3, 4]. In-office vitrectomy either requires an in-office full surgical setup with operating microscope [5], or is performed under indirect ophthalmoscopy with an inverted view [6–9]. This is challenging even for the most experienced surgeons and can be tedious to set up in-office. Therefore, there has been interest in developing a limited vitreous biopsy technique that can be performed in-office, with the ability to perform a limited number of cuts while withdrawing vitreous to reduce the rate of a dry tap. Previously, we demonstrated that it is possible to perform diagnostic vitreous biopsy in the clinic using a trocar/cannula system with a standard 25 or 27-gauge vitreous cutter (Alcon, Fort Worth, TX), actuated with two separate syringes [10]. However, this requires opening a full surgical pack for the cutter, a viscous fluid control module for a fitting, and multiple additional assembly steps; this can be onerous to arrange in the office, limiting use to motivated clinicians. Here, we are presenting a novel, simplified technique for vitreous tap using a vitreous cutter provided in a single peel pack, actuated by a single syringe with minimal assembly prior to use, making it more accessible for clinicians than prior techniques.

## Methods

**Fig. 1:**
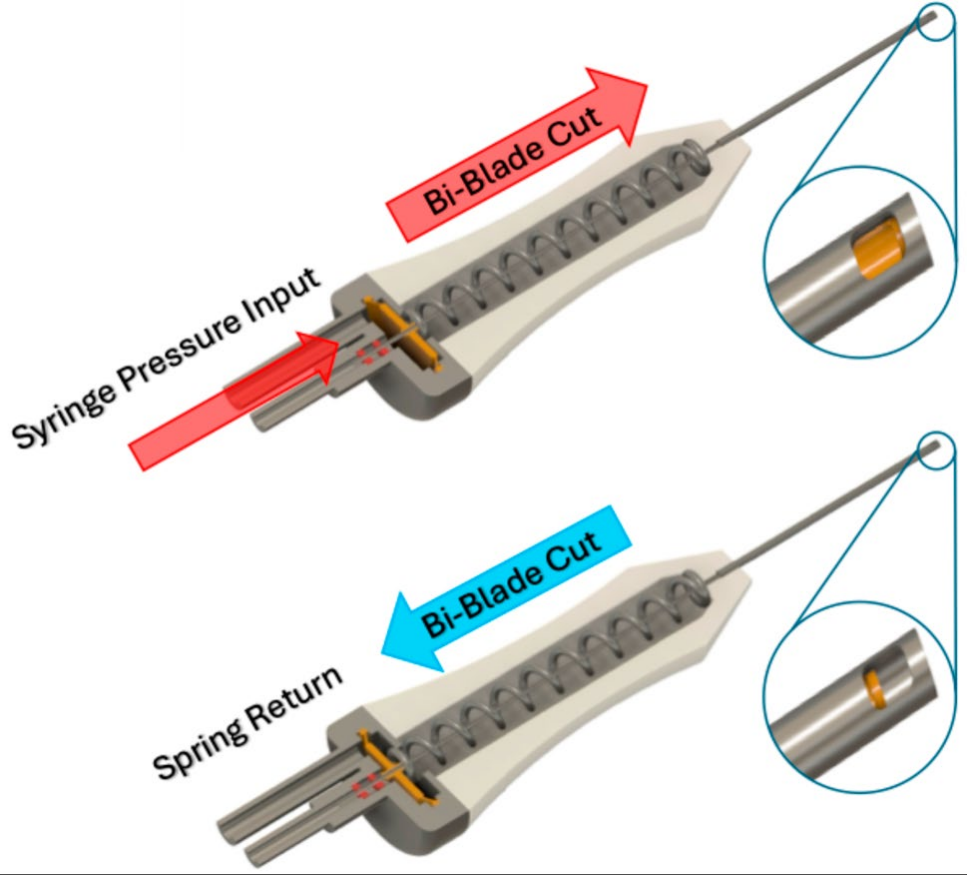
Top: a pressure pulse from the syringe causes the diaphragm of the cutter to advance, closing the tip and causing a cut on the upstroke. Bottom: once pressure on the syringe is released, the spring returns the cutter and it cuts on the downstroke.

The vitreous biopsy peel pack technique involves using a single actuation cutting technique, actuated by a syringe held by an assistant. As shown in the supplemental animation and in Fig. 1, pressure pulses from a surgical console - or in this case from a syringe - increase pressure on a diaphragm, which then causes the cutter to close and cut. When pressure is released, the spring opens the cutter again. With a Bi-Blade cutter, cutting occurs on both closing and opening of the cutter.

**Fig. 2:**
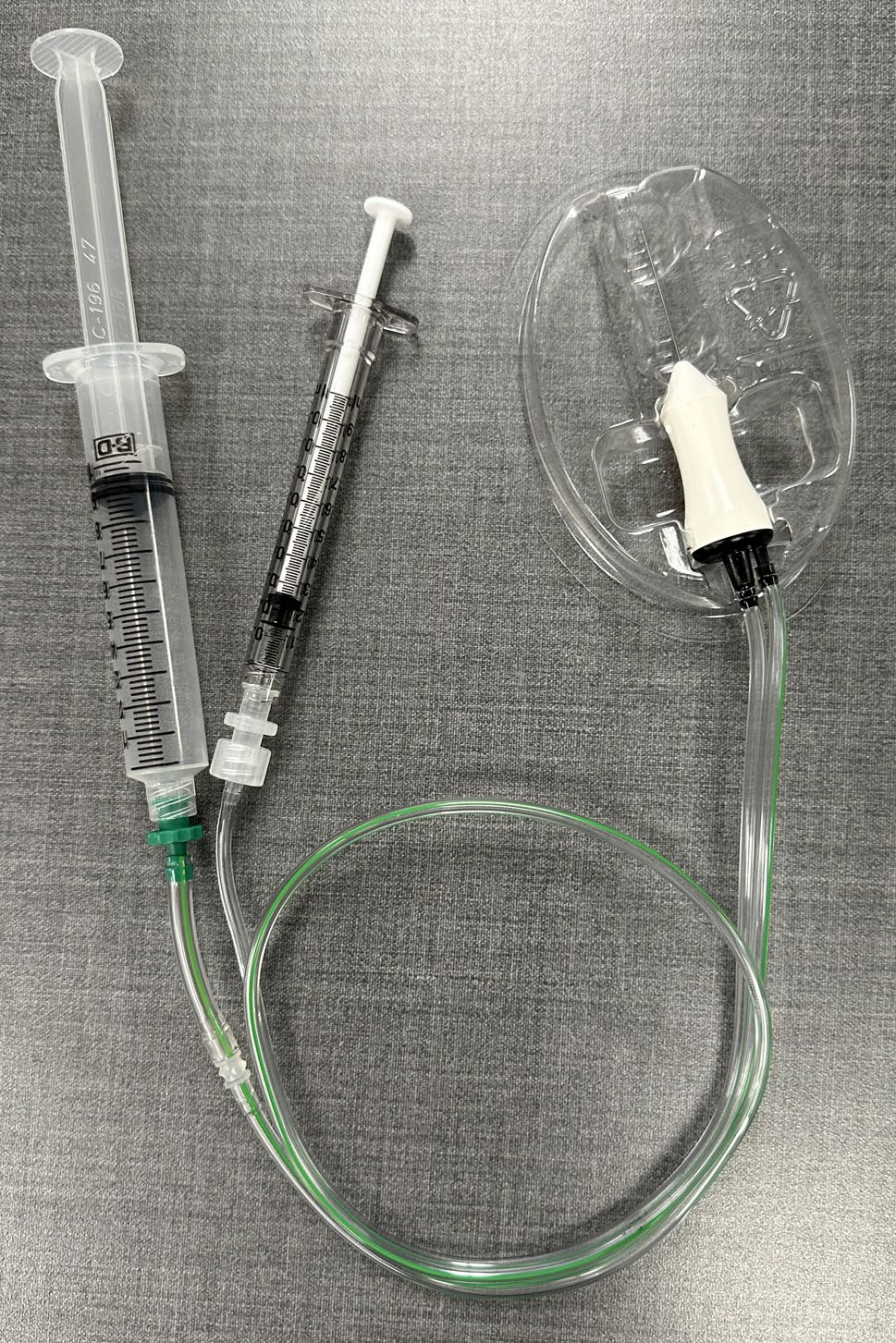
Assembled cutter for vitreous biopsy. The 25-gauge Bi-Blade MIDLabs cutter has a 1 mL syringe attached to the aspiration line (clear). The drive line (green) has been cut short with the Luer fitting re-attached. A 10 mL syringe has been attached to the drive line.

A 25-gauge trocar/cannula system along with sterile jeweler’s forceps are placed on the sterile field. A 1 mL syringe of sterile balanced salt solution, along with any additional injectables (antibiotics, antifungals, or antivirals) are prepared on 30-gauge 1/2 inch needles. The components of the cutter are assembled in the sterile field. A 2740BB MIDLabs (San Leandro, CA) vitreous cutter is opened from a sterile peel pack. A sterile 1 mL syringe is attached to the proximal Luer Lock fitting from the central aspiration line and the distal line is removed. The barbed Luer Lock fitting from the drive line is manually removed from the end of the line, the line is cut with sterile scissors before the transition to smaller diameter, and the barbed Luer Lock is placed closer to the cutter. A sterile 10 mL syringe is brought to the 10 mL mark and attached to the drive line (Fig. 2). Before use, an assistant actuates the cutter by applying and releasing pressure to the 10 mL syringe. The surgeon is able to feel the cutter opening and closing when holding the cutter.

The eye is anesthetized with proparacaine and subconjunctival 2% lidocaine, opened with a speculum and prepared with betadine solution. The 25-gauge cannula is placed 3.5–4.0 mm from the limbus in either the inferotemporal or superotemporal quadrant based on surgeon preference [11]. The vitreous cutter is inserted into the mid-vitreous with care to avoid the lens. The assistant actuates the cutter by applying and releasing pressure on the 10 mL syringe, while the surgeon aspirates a 0.5 mL vitreous sample. The cutter is removed and residual vitreous from the line is aspirated into the syringe.

Intravitreal antibiotics/antifungals/antivirals are injected as deemed necessary through the cannula. If the eye remains hypotonous, sterile balanced salt solution can be injected. The cannula is removed with the jeweler’s forceps.

During the process, it is important to account for the fact that the aspiration line has 0.7 mL of fluid before it reaches the aspiration syringe. In fact, 0.5 mL fills a little over half of the aspiration line. This vitreous is aspirated into the syringe before discarding the cutter. The authors recommend having a syringe with sterile balanced salt solution prepared for injection to manage post-sample hypotony. Although, in the majority of cases performed by the authors, this has not been required.

## Results

The vitreous cutter peel pack technique has been used as a viable alternative method of vitreous biopsy in clinic.

## Discussion

Here, we describe a technique that can obtain higher volume vitreous samples reliably through limited vitreous cutting. This technique is meant to improve the ability of the clinician to accurately diagnose and manage disease promptly at the bedside. The cutter used in this procedure (MIDLabs, San Leandro, CA) was selected because it is available in an individual peel pack and has Luer Lock fittings to directly allow a single syringe to be attached for actuation.

Our initial work used a dual actuation cutter (Alcon, Ft. Worth) as it is provided with many of the commonly used vitrectomy machines and is widely available in many operating suites [10]. In this work, a cutter not as commonly used for standard vitrectomy surgery was specifically sourced from the vendor (MIDLabs, San Leandro, CA) to simplify assembly.

Potential advantages of this technique include that it is a slow, controlled method to remove vitreous by taking discrete bites of vitreous with the cutter. The flow of vitreous can be observed in the aspiration line by the clinician as it is slowly aspirated between cuts. Physical cutting of the vitreous may reduce clogging as seen in needle biopsy and therefore reduce the chance of a dry tap. Portable vitrectomy machines have been used for a similar purpose and may be able to remove higher volumes of vitreous. However, these require additional capital equipment, maintenance, and working inverted using indirect ophthalmoscopy for visualization [5–7].

Another option is vitreous biopsy using automated vitrectomy in the operating room, where larger vitreous samples can be collected, there is a superior level of control of intraocular pressure from active infusion, and direct visualization may be possible [12, 13]. However, active infusion risks diluting the sample or is not switched on until dry sample is taken, causing hypotony prior to re-inflation of the eye. While direct visualization may be possible for uveitis and lymphoma, visualization is often very poor in post operative endophthalmitis. Despite potential advantages of pars plana vitrectomy, studies have found conflicting benefits of vitrectomy versus tap and inject for endophthalmitis, with many practitioners performing vitreous biopsy in the clinic [14].

Hence, while automated vitrectomy in the operating room remains the gold standard, vitreous biopsy can be deployed if the patient is too sick for surgery, the operating room is unavailable or will lead to unacceptable delays. Further, many practitioners may choose to perform a full vitrectomy as a therapeutic intervention following diagnostic biopsy.

Pfhaler et al. found 0.05–0.1 mL vitreous taps were well tolerated in a prospective study of 578 vitreous taps prior to intravitreal injection. The majority of patients were being managed for age-related macular degeneration, proliferative diabetic retinopathy, and vein occlusions. The dry tap rate was found to be 4.8% [4]. This low dry tap rate may be due to the average age of 77 years as well as lack of inflammation and infection, which cause vitreous condensation and fibrin. Ghodasra et al. evaluated office-based needle biopsy of 0.2 mL with a 25-gauge needle and surgical based vitreous biopsy for cytokine analysis and found 12% of samples from office-based needle biopsy were inadequate for analysis due to low volume or dry tap [15]. AlBloushi et al. retrospectively evaluated 217 patients with vitreous and aqueous tap for acute endophthalmitis and found a 32% positive culture rate from vitreous tap using 27 − 22 gauge needles [16]. Lobo et al. used a 21-gauge needle vitreous biopsy to help differentiate infectious, inflammatory, and malignancy with a successful sample rate of 92% [17]. Of 26 patients with suspected primary intraocular lymphoma, the diagnosis was confirmed in 5 patients with needle aspiration, while 2 required additional procedures for diagnosis confirmation. Vahedi et al. evaluated using a trocar cannula technique vs. standard vitreous tap in 18 patients and had a dry tap rate between 22 and 44% [18]. Giovinazzo et al. evaluated needle-based biopsy versus trocar/cannula biopsies in acute endophthalmitis and found a dry tap rate of 34% when using a needle and 0% with a trocar/cannula technique [3]. Further studies are needed for our technique to determine yield.

### Limitations

Limitations of this work include that the technique is not performed under direct visualization. Thus, there are potential risks which include extending the vitreous cutter deeply into the eye, direct contact with the lens, contact with the retina, or removal of a high volume of sample and possible hypotony. These are the same risks involved with other vitreous sampling techniques; however it remains important to evaluate for retinal detachment or choroidals prior to use with b-scan, to avoid inserting the cutter too deep into the eye, and to angle the cutter to avoid lens contact in phakic patients.

As this is a new technique, further evaluation is required to understand the overall safety and diagnostic yield relative to standard 25-gauge needle tap. At this point, the vitreous biopsy peel pack technique has been performed on a 30-year old patient to help differentiate between immune-related adverse events (irAE) and infectious process and a 61 year old patient with fulminant endophthalmitis within a single institution. In both cases with this technique, between 0.4 mL to 0.5 mL of vitreous was safely removed without requiring additional infusion of balanced salt solution. Cut rates for both patients were determined by the assistant actuating the syringe with cut rates of approximately 60 cuts per minute. Further work will include evaluation with more subjects and a control group with short and long-term complication rates to evaluate the technique’s overall safety and efficacy.

## Conclusion

In conclusion, we report a simpler technique and easier setup process of using individually supplied disposable vitrectomy cutter peel packs to perform vitreous biopsy in-clinic with a 25-gauge trocar/cannula system. This is expected to increase sample yield for in-office procedures without requiring additional capital equipment.

## Electronic supplementary material

Below is the link to the electronic supplementary material.

Supplementary Material 1

## Data Availability

No datasets were generated or analysed during the current study.
